# Is non-HDL-cholesterol a better predictor of long-term outcome in patients after acute myocardial infarction compared to LDL-cholesterol? : a retrospective study

**DOI:** 10.1186/s12872-016-0450-9

**Published:** 2017-01-05

**Authors:** Wanwarang Wongcharoen, Satjatham Sutthiwutthichai, Siriluck Gunaparn, Arintaya Phrommintikul

**Affiliations:** Department of Internal Medicine, Faculty of Medicine, Chiang Mai University, Chiang Mai, Thailand

**Keywords:** Non-HDL-cholesterol, LDL-cholesterol, Acute myocardial infarction, Major adverse cardiovascular events

## Abstract

**Background:**

It has recently been shown that non-high density lipoprotein cholesterol (non-HDL-C) may be a better predictor of cardiovascular risk than low density lipoprotein cholesterol (LDL-C). Based on known ethic differences in lipid parameters and cardiovascular risk prediction, we sought to study the predictability of attaining non-HDL-C target and long-term major adverse cardiovascular event (MACE) in Thai patients after acute myocardial infarction (AMI) compared to attaining LDL-C target.

**Methods:**

We retrospectively obtained the data of all patients who were admitted at Maharaj Nakorn Chiang Mai hospital due to AMI during 2006–2013. The mean non-HDL-C and LDL-C during long-term follow-up were used to predict MACE at each time point. The patients were classified as target attainment if non-HDL-C <100 mg/dl and/or LDL-C <70 mg/dl. The MACE was defined as combination of all-cause death, nonfatal coronary event and nonfatal stroke.

**Results:**

During mean follow-up of 2.6 ± 1.6 years among 868 patients after AMI, 34.4% achieved non-HDL-C target, 23.7% achieved LDL-C target and 21.2% experienced MACEs. LDL-C and non-HDL-C were directly compared in Cox regression model. Compared with non-HDL-C <100 mg/dl, patients with non-HDL-C of >130 mg/dl had higher incidence of MACEs (HR 3.15, 95% CI 1.46–6.80, *P* = 0.003). Surprisingly, LDL-C >100 mg/dl was associated with reduced risk of MACE as compared to LDL <70 mg/dl (HR 0.42, 95% CI 0.18–0.98, *p* = 0.046) after direct pairwise comparison with non-HDL-C level.

**Conclusions:**

Non-attaining non-HDL-C goal predicted MACE at long-term follow-up after AMI whereas non-attaining LDL-C goal was not associated with the higher risk. Therefore, non-HDL-C may be a more suitable target of dyslipidemia treatment than LDL-C in patients after AMI.

**Electronic supplementary material:**

The online version of this article (doi:10.1186/s12872-016-0450-9) contains supplementary material, which is available to authorized users.

## Background

It is well-established that low-density lipoprotein cholesterol (LDL-C) is an important risk factor for coronary heart disease. The international guidelines recommend LDL-C as a primary target of therapy in persons with hypercholesterolemia and non-high-density lipoprotein cholesterol (non-HDL-C) as a secondary target of therapy in persons with triglyceride at least 200 mg/dl [1, 2].

Previous epidemiologic studies have shown that non-HDL-C is more strongly associated with coronary heart disease risk than LDL-C [3–5]. In addition, recent post-hoc analyses have demonstrated that the on-treatment level of non-HDL-C is more closely associated with cardiovascular outcome than levels of LDL-C. These findings suggest that the residual risk after lipid-lowering treatment may be better quantified by non-HDL-C than by LDL-C [6].

A number of studies have shown that there are ethnic differences in risk prediction of coronary artery disease. The Framingham prediction model accurately predicts the coronary artery disease risk among Caucasians and blacks living in the United States, however, it overestimates the risk in South-East Asians [7]. In addition, the data from the Electricity Generating Authority of Thailand (EGAT) cohort study showed that only HDL-C was negatively associated with cardiovascular disease mortality [8]. However, triglyceride and LDL-C were not associated with cardiovascular death in Thai population, which was inconsistent with previous studies in other ethnic populations [9]. Although a growing body of evidence supports that non-HDL-C is superior to LDL-C in predicting long-term cardiovascular risk, there is limited data in South-East Asian population.

Based on known ethic differences in lipid parameters and cardiovascular risk prediction, we sought to study the predictability of attaining non-HDL-C target and long-term cardiovascular outcome in Thai patients after acute myocardial infarction (AMI) compared to attaining LDL-C target.

## Methods

### Studied population

This is a retrospective cohort study. The 868 patients admitted in Maharaj Nakorn Chiang Mai hospital with a diagnosis of AMI during a period of 2006–2013 were enrolled into the study. The patients who did not have lipid profile data during the treatment and patients who had a follow-up period less than 3 months were excluded from the study.

The primary objective of the study was to assess the predictability of attaining non-HDL-C goal and LDL-C goal on the long-term major adverse cardiovascular events (MACE) occurrence in patients after AMI. The secondary objective of the study was to identify other predictors of long-term MACE occurrence in patients after AMI.

The study protocol was approved by the Medical Ethics Committee of Faculty of Medicine, Chiang Mai University.

### Definitions

▪ Acute myocardial infarction:

- Typical rise and/or fall of biochemical markers of myocardial necrosis with at least one of the followings:
  - Ischemic symptoms
  - Development of pathologic Q waves in the ECG
  - Electrocardiographic changes indicative of ischemia (ST-segment elevation or depression)
  - Imaging evidence of new loss of viable myocardium or new regional wall motion abnormality

▪ Major adverse cardiovascular outcomes

- Defined as a composite outcome of all-cause death, myocardial infarction, stroke and cardiovascular hospitalization.

▪ Achieved target of non-HDL-C and LDL-C

- The patients were classified as achieving target if the mean non-HDL-C was less than 100 mg/dL and/or the mean LDL-C was less than 70 mg/dl.
- The patients were classified as failure to achieve target if the mean non-HDL-C was more than 130 mg/dL and/or the mean LDL-C was more than 100 mg/dl.

### Data collection

The medical records of patients diagnosed with AMI and admitted in the hospital during 2006–2013 were reviewed. Data from medical record included baseline characteristic, cardiovascular risk, diagnostic data of AMI, lipid parameters, and MACE outcomes. Lipid parameters used in the data analysis included LDL-C and non-HDL-C. In this analysis, we examined the relationship between the lipid parameters at admission, the mean lipid parameters during long-term follow-up and cardiovascular outcomes (Additional file 1).

### Statistical analysis

Differences between continuous variables were assessed using an unpaired 2-tailed t test for normally distributed continuous variables and the Mann-Whitney test for skewed variables. Proportions were compared by Chi-square test or Fisher exact test when appropriate. The recurrence— free survival curve was plotted via the Kaplan-Meier method with the statistical significance examined by the log-rank test. Multivariate Cox regression analysis was performed for variables with a *p* value of less than 0.1 in univariate analysis. All statistical significances were set at *p* value <0.05 and all statistical analyses were carried out by SPSS 17.0 (SPSS Inc. USA).

## Results

Between 2006 and 2013, there were 868 patients admitted due to AMI and enrolled into the study. The mean age was 63 ± 11 years. There was higher prevalence of male (62%) in this population. Majority of patients presented with acute ST-segment elevation MI (STEMI). There were 20.9% presented with non-ST-segment elevation MI (non-STEMI) and only 1.5% presented with unstable angina. Among 674 patients who had ST-elevation MI, 399 (59.2%) patients underwent primary PCI, 222 (32.9%) patients received fibrinolytic therapy while 53 (7.9%) did not receive reperfusion therapy. All patients had been receiving antiplatelets. Beta-blocker, angiotensin converting enzyme inhibitor (ACEI) and angiotensin receptor blocker (ARB) had been prescribed in 79.7%, 64.9% and 28.0% of the patients, respectively.

During a mean follow-up of 2.6 ± 1.6 years, patients had lipid parameter evaluation according to their physicians and the mean interval of lipid parameters follow-up was 6.5 ± 7.5 months. Among 868 patients, 23.7% achieved LDL-C target and 34.4% achieved non-HDL-C target. Table 1 shows the baseline characteristics of the three groups as defined by their LDL-C level: <70 mg/dl, 70–100 mg/dl, and >100 mg/dl. Table 2 shows the baseline characteristics of the three groups as defined by their non-HDL-C level: <100 mg/dl, 100–130 mg/dl, and >130 mg/dl. The patients who attained either LDL-C target or Non-HDL-C target were significantly older and had higher prevalence of chronic kidney disease, compared to those who did not attain the corresponding target. In addition, the baseline LDL-C and baseline non-HDL-C were significantly lower in those with attaining either LDL-C or non-HDL-C target. Statin had been prescribed in 93.0% of the patients, similarly across different LDL-C and non-HDL-C groups. Ezetimibe had been prescribed in addition to statin in 3.9% of the patients. There was a higher proportion of patients with mean LDL-C >100 mg/dl receiving ezetimibe compared to those with lower LDL-C level (Tables 1 and 2).

**Table 1:** Baseline characteristics of patients among different mean LDL-C groups

Parameter	LDL-C <70 mg/dl (*N* = 206, 24% )	LDL-C 70–100 mg/dl (*N* = 405, 46%)	LDL-C >100 mg/dl (*N* = 257, 30%)	*P*-value
Age (years)	66.0 ± 11.2	63.3 ± 11.0	60.7 ± 11.1	<0.001
Male	65.6%	62.2%	58.7%	0.312
Body mass index (kg/m^2^)	22.5 ± 4.4	22.8 ± 4.5	23.5 ± 5.2	0.122
LVEF (%)	50.0 ± 13.8	50.1 ± 13.8	50.9 ± 14.0	0.772
Creatinine (mg/dl)	2.2 ± 10.2	1.6 ± 2.2	1.7 ± 7.6	0.492
Hemoglobin (g/dl)	12.5 ± 2.8	12.4 ± 2.1	12.7 ± 2.0	0.224
Baseline LDL-C (mg/dl)	97.3 ± 38.5	111.2 ± 39.0	135.3 ± 44.0	<0.001
Baseline non-HDL-C (mg/dl)	121.9 ± 39.5	137.5 ± 43.9	162.7 ± 48.6	<0.001
Baseline HDL-C (mg/dl)	40.4 ± 11.3	39.2 ± 11.6	40.4 ± 9.6	0.322
Smoking	38.0%	35.6%	37.4%	0.714
Hypertension	61.2%	57.3%	53.3%	0.222
Dyslipidemia	29.6%	30.4%	38.1%	0.978
Diabetes mellitus	30.1%	27.4%	24.1%	0.348
Chronic kidney disease	9.7%	7.9%	3.9%	0.039
History of CAD	10.2%	8.9%	12/1%	0.419
History of stroke	4.9%	4.2%	4.7%	0.921
STEMI	78.0%	80.0%	73.7%	0.190
Statin	92.2%	93.8%	92.2%	0.65
Ezetimibe	2.4%	2.5%	7.4%	0.003

*CAD* coronary artery disease, *LVEF* left ventricular ejection fraction, *STEMI* ST elevation myocardial infarction

**Table 2:** Baseline characteristics of patients among different mean non-HDL-C groups

Parameter	Non-HDL-C <100 mg/dl (*N* = 299, 34%)	Non-HDL-C 100–130 mg/dl (*N* = 333, 38%)	Non-HDL-C >130 mg/dl (*N* = 236, 27%)	*P*-value
Age (years)	65.9 ± 11.0	62.5 ± 11.1	60.6 ± 11.0	<0.001
Male	66.2%	60.1%	59.3%	0.173
Body mass index (kg/m^2^)	22.5 ± 4.5	23.0 ± 5.5	23.4 ± 3.9	0.252
LVEF (%)	49.9 ± 14.4	50.8 ± 13.0	50.0 ± 14.2	0.715
Creatinine (mg/dl)	2.1 ± 8.7	1.7 ± 6.8	1.4 ± 2.2	0.528
Hemoglobin (g/dl)	12.5 ± 2.5	12.5 ± 2.1	12.6 ± 2.0	0.681
Baseline LDL-C (mg/dl)	101.1 ± 38.5	116.1 ± 39.6	131.7 ± 46.1	<0.001
Baseline non-HDL-C (mg/dl)	122.1 ± 38.7	142.1 ± 42.5	164.3 ± 51.4	<0.001
Baseline HDL-C (mg/dl)	39.7 ± 11.3	40.2 ± 11.0	39.8 ± 10.5	0.822
Smoking	37.8%	35.0%	38.6%	0.850
Hypertension	57.9%	59.2%	53.8%	0.477
Dyslipidemia	25.8%	34.2%	38.6%	0.0.05
Diabetes mellitus	27.1%	26.4%	28.0%	0.920
Chronic kidney disease	9.0%	5.4%	7.2%	0.210
History of CAD	8.4%	9.0%	14.0%	0.07
History of stroke	5.0%	3.9%	4.7%	0.788
STEMI	79.3%	79.9%	72.5%	0.181
Statin	93.0%	92.8%	93.2%	0.981
Ezetimibe	3.3%	2.4%	6.8%	0.024

*CAD* coronary artery disease, *LVEF* left ventricular ejection fraction, *STEMI* ST elevation myocardial infarction

During follow up, total MACE occurred in 184 (21.2%) patients. There were 25.2, 19.0 and 21.4% of patients developed MACEs in group of LDL-C <70 mg/dl, LDL-C 70–100 mg/dl and LDL >100 mg/dl respectively. There were 20.1, 18.9 and 25.8% of patients developed MACEs in group of non-HDL-C <100 mg/dl, non-HDL-C 100–130 mg/dl and non-HDL-C >130 mg/dl respectively.

We first examined the predictability of LDL-C and long-term MACEs and the predictability of non-HDL-C and long-term MACEs individually. After cox regression analysis adjusted with age, gender, comorbidities, and baseline lipid parameters, patients with LDL70–100 mg/dl and LDL >100 mg/dl had neutral risk of long-term MACEs with the adjusted HR of 0.98 (95% CI 0.57–1.60) and 1.02 (95% CI 0.56–1.84), compared to patients with LDL-C <70 mg/dl. On the contrary, we found that non-attaining non-HDL-C goal could predict the risk of long-term MACEs. Compared to patients with non-HDL-C <100 mg/dl, those with non-HDL-C 100–130 mg/dl had a non-significantly increased risk of MACEs (adjusted HR 1.10; 95% CI 0.65–1.85) and those with non-HDL-C >130 mg/dl had significantly higher risk of long-term MACEs (adjusted HR 1.75; 95% CI 1.02–3.00, *P* = 0.04).

**Fig. 1:**
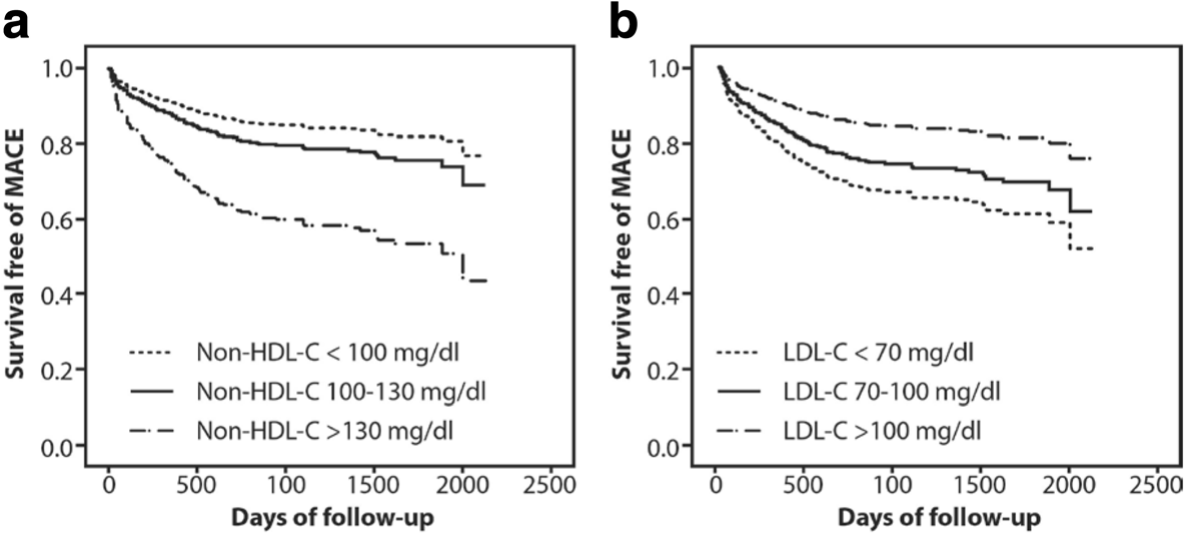
Time to first major adverse cardiovascular events. Cox regression analysis of time to the first major adverse cardiovascular events after direct pairwise comparison of LDL-C and non-HDL-C. **a** Time to first major adverse cardiovascular events among three different non-HDL-C groups. **b** Time to first major adverse cardiovascular events among three different LDL-C groups

Then, we directly compared the strengths of the association of LDL-C and non-HDL-C with long-term MACEs by including LDL-C and non-HDL-C in the Cox model simultaneously. We demonstrated the stronger association between non-HDL-C and long-term MACEs after the direct comparison with LDL-C. Compared to non-HDL-C <100 mg/dl, patients with non-HDL-C >130 mg/dl tripled the risk of long-term MACEs (adjusted HR 3.15, 95% CI 1.46–6.80, *P* = 0.003). Conversely, LDL-C >100 mg/dl was inversely associated with the long-term MACEs when compared to LDL <70 mg/dl (adjusted HR 0.42, 95% CI 0.18 – 0.98, *p* = 0.046) (Table 3 and Fig. 1). With this regard, we demonstrated that for a given non-HDL-C level, an increase in LDL-C was associated with a reduced risk of long-term MACEs. Due to the possibility of the correlation between LDL-C and non-HDL-C, we performed the collinearity analysis for variance inflation factor (VIF) and demonstrated no collinearity between LDL-C and non-HDL-C.

**Table 3:** Individual relationships and direct pairwise comparison of LDL-C and non-HDL-C and time to the first major adverse cardiovascular events

Variables	Adjusted hazard ratio^a^	95% CI	*P*-value	Adjusted hazard ratio^b^	95% CI	*P*-value
LDL-C <70 mg/dl	1.00			1.00		
LDL-C 70–100 mg/dl	0.98	0.57–1.60	0.934	0.74	0.39–1.40	0.350
LDL-C >100 mg/dl	1.02	0.56–1.84	0.956	0.42	0.18–0.98	0.046
Non-HDL-C <100 mg/dl	1.00			1.00		
Non-HDL-C 100–130 mg/dl	1.10	0.65–1.85	0.715	1.40	0.74–2.65	0.304
Non-HDL-C >130 mg/dl	1.75	1.02–3.00	0.04	3.15	1.46–6.80	0.003

^a^Individual relationships of LDL-C, non-HDL-C and time to the first major adverse cardiovascular events calculated by a Cox proportional hazard model with adjustment for age, sex and comorbidities

^b^Direct pairwise comparison of LDL-C, non-HDL-C and time to the first major adverse cardiovascular events calculated by a Cox proportional hazard model with adjustment for age, sex and comorbidities

The association between the incidence of MACEs and the frequency that the patients achieved LDL or Non-HDL targets during the long-term follow-up were also examined. The patients with long-term MACEs had similar percentage of dosage on Non-HDL target compared to those without long-term MACEs (31.0 ± 31.4% vs. 29.1 ± 32.3%, *P* = 0.496). On the contrary, patients with long-term MACEs had higher percentage of dosage on LDL target than those without long-term MACEs (27.2 ± 28.8% vs. 20.6 ± 28.8%, *P* = 0.006).

Furthermore, the other independent predictors of long-term MACEs were observed. After adjustment with covariates, we found that age, the lower left ventricular ejection fraction (LVEF) and non-STEMI were also the independent predictors of long-term MACE in this population.

## Discussion

### Main findings

Our study demonstrated that (1) relatively low proportion of patients after AMI achieved lipid treatment goal. Only 24% and 34% of patients after AMI attained LDL-C goal and non-HDL-C goal during long-term follow-up. (2) After cox regression analysis, we demonstrated that non-attaining non-HDL-C goal was associated with higher risk of long-term MACE, whereas the non-attaining LDL-C goal was not associated with the increased risk of long-term MACE. (3) The other independent predictors of long-term MACE were age, impaired LVEF and non-STEMI.

Pharmacologic lipid management after AMI is crucial for secondary prevention of cardiovascular events [10–12]. We observed that the low proportion of our studied population could attain lipid target goal during long-term follow-up. Therefore, aggressive lipid-lowering treatment should be reinforced in order to achieve the therapeutic target which may lead to the lower risk of long-term MACE in this high-risk population.

Non-HDL-C composites of all atherogenic apolipoprotein B-containing lipoproteins, including LDL-C, very low-density lipoprotein cholesterol (VLDL-C), intermediate-density lipoprotein cholesterol (IDL-C), lipoprotein(a), chylomicrons, and chylomicron remnants [4]. Therefore, non-HDL-C is a more comprehensive measure of atherogenic particles than LDL-C.

Previous studies have investigated the relationships between LDL-C or non-HDL-C and the risk of coronary heart disease. The Health Professionals Follow-up Study showed that non-HDL-C was more strongly associated with coronary heart disease risk than LDL-C [5]. Similarly, the Framingham Heart Study showed that at every non-HDL-C level, the concentration of LDL-C was not associated with the risk for coronary heart disease. On the contrary, at every LDL-C level, a strong positive and graded association between non-HDL-C and risk of coronary heart disease was observed [13]. In addition, Liu et al. showed that coronary heart disease risk in patients with diabetes was significantly associated with increasing non-HDL-C, but not with increasing LDL-C. They concluded that among patients with diabetes, non-HDL-C was a stronger predictor of coronary heart disease death than LDL-C [3].

A number of studies have shown that there are ethnic differences in risk prediction of coronary artery disease as well as response to treatment [7–9, 14]. In the present study, we demonstrated that Thai patients who did not attain non-HDL-C goal had higher risk of long-term MACE, compared to those who attained non-HDL-C goal. Our findings were in accordance with other studies of western population. Interestingly, we observed that non-attaining LDL-C goal did not correlate with the long-term risk of MACEs. Counter intuitively, patients with mean LDL-C >100 mg/dl had fewer cardiovascular events than those with mean LDL-C <70 mg/dl after direct pairwise comparison with non-HDL-C. This indicated that for a given non-HDL-C level, an increase in LDL-C was associated with a reduced risk of long-term MACEs. It is well-established that the large LDL particle is associated with the lower risk of cardiovascular events than the small dense LDL particle [15]. The inverse association between LDL-C and long-term MACEs observed in the present study may be explained by the fact that patients with higher LDL-C level had larger LDL particle size than those with lower LDL-C level after adjustment with non-HDL-C level. Previous study by Kastelein and colleagues reported similar findings that LDL-C level after statin treatment was inversely associated with adverse cardiovascular outcome after direct pairwise comparison with non-HDL-C level [6].

We demonstrated that non-HDL-C was a more accurate predictor of long-term MACEs than LDL-C in our population after AMI. As the non-HDL-C can be simply calculated by subtracting HDL-C from total cholesterol, therefore, measurement of non-HDL-C incurs no additional cost. With these regards, non-HDL-C should favorably be used as a therapeutic target in the treatment of dyslipidemia in patients after AMI. Our findings support the recommendations from the international atherosclerosis society and national institute of health and care excellence (NICE) which favor the use of non-HDL-C over LDL-C as targets of therapy [16, 17].

## Conclusions

Non-attaining non-HDL-C goal was associated with higher risk of long-term MACEs. However, we did not find the correlation between non-attaining LDL-C goal and the increased risk of MACEs. Therefore, non-HDL-C may be a more suitable target of dyslipidemia treatment than LDL-C in patients after AMI. In addition, we demonstrated that only small proportion of patients after AMI could achieve lipid targets during long-term follow-up. More aggressive lipid-lowering strategy should be implemented aiming to reduce the risk of cardiovascular outcome in this high-risk population.

## Additional file

Additional file 1: Non HDL-C vs LDL-C and long term outcomes. The dataset for the non-HDL-C and LDL-C on long term cardiovascular outcomes after AMI analysis. (XLS 338 kb)

## Abbreviations

ACEI: Angiotensin converting enzyme inhibitor
AMI: Acute myocardial infarction
ARB: Angiotensin receptor blocker
CAD: Coronary artery disease
LDL-C: Low-density lipoprotein cholesterol
LVEF: Left ventricular ejection fraction
MACE: Major adverse cardiovascular events
Non-HDL-C: Non-high-density lipoprotein cholesterol
Non-STEMI: Non ST-segment elevation myocardial infarction
STEMI: ST-segment elevation myocardial infarction
